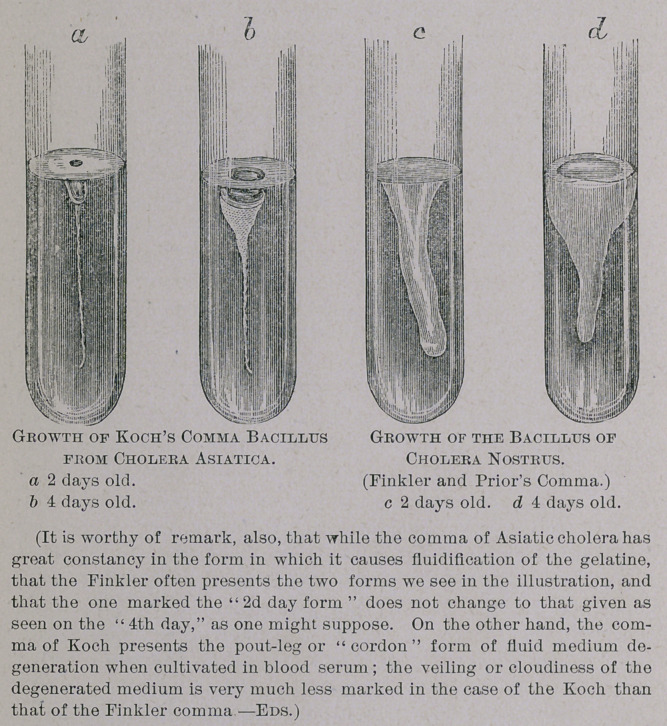# Robert Koch, the Conductor of the German Cholera Commission, and His Method of Bacteria Cultivation

**Published:** 1885-04

**Authors:** Albert Johne

**Affiliations:** The Royal Veterinary Institute, Dresden


Robert Koch.

// THE JOURNAL

OF

COMPARATIVE MEDICINE AND SURGERY.

VOL. VI. APRIL, 1885. No. 2

### ORIGINAL COMMUNICATIONS.

### Art. IX.—ROBERT KOCH,

### The Conductor of the German Cholera Commission.—His Method of Bacteria Cultivation.

BY PROF. DR. ALBERT JOHNE,
of the Royal Veterinary Institute, Dresden.

( *Written especially for the* Journal of Comparative Medicine and Surgery).

There are few among the names that give lustre to the horizon of scientific research which shine with greater brilliancy than that of the world renowned investigator in the field of bacteriology, especially with reference to cholera, "Geheim Regierungsrath," Dr. Robert Koch, who is also an active member of the Imperial Board of Health of Germany. It cannot but be of interest to the readers of the Journal to learn something of the life of Koch, after having been favored with a sketch of that of his great French contemporary Pasteur in the last number.

Koch was born at Clausthal (in the Hartz Mountains) in the year 1843, and studied medicine from 1862—1866 at Göttingen and Berlin. In the summer of 1866 we find him acting as assistant at the General Hospital of the City of Hamburg; from thence he began the life of a general practitioner, first at

Langenhogen, in the vicinity of Hanover, and later at Rackwitz, in the Province of Posen, and finally in a small city known as Wallstein in the same Province. In 1872 he was appointed to the position of "Kreis-physicus" for the last named locality. Here in Wallstein Koch laid the foundation of those researches which threaten to almost revolutionize many of the ideas of General Pathology, and this while busily engaged in a constantly augmenting practice which was largely surgical in its character.

Here it was that he made those detailed studies into the etiology of anthrax and the biology of Bacillus Anthracis—"Cohn. Biologie der Pflanzen," II. p. 277—which revealed to us almost the entire life-history of this parasite; although it had been familiar to observers for nearly a quarter of a century. By means of Koch's investigations in this direction, we have become enabled to more successfully combat the extension of this fell disease among our animal population. Not content with such brilliant success in one direction, our investigator at once sought for other fields in which to make himself useful to humanity, and we find him busied in those wonderful researches, with which we are all now so familiar, upon disease as caused by infection from wounds. (Etiology der Wound-infections Krankheiten, Leipzig, 1876.) In these studies he confirmed the observations made by Klebs, Recklinghausen, Waldeyer and Birch-Hirschfeld, with reference to the occurrence of micrococci in connection with pyæmia and puerperal fever, as well as in pyæmia in the rabbit, and also in other accidental diseases of traumatic origin among animals, progressive necrosis of the tissues, progressive development of abcesses, septicæmia and erysipelas in rabbits, and the disease which has become somewhat noted through these researches, septicæmia in the mouse. By direct experiment he proved that all these diseases were due to the direct introduction of micrococci into the organism, thus giving direct scientific proof to the conclusions which Mr. Lister has so ably drawn from and supported by practical experience.

It was but natural that work of such an exact and scientific character, as well as great practical importance, should finally attract the attention of the medical world, as well as the government, to the humble "Kreis-physicus" at Wallstein. In

1879 Koch was promoted to "Physicus," and removed to the more favorable city of Breslau, but the circumstances here do not seem to have suited him, for he soon returned to his former residence, fortunately, however, but for a short time. In 1880 he was made an ordinary member of the Imperial Board of Health, of which we can truly say he has been the most important member, as well as worker.

Since his connection with this important branch of the government we have become familiar with a succession of the most brilliant researches in the field of Preventive Medicine which ever issued from the brain and hands of mortal man, as may be seen by reference to the two volumes issued by the Board under the title of "Mittheilungen des Kaiserlichen Gesenedheitzamtes, 1881-3." In the first volume the reader is introduced to the peculiar method of bacteria culture, which have been developed by the master of this art in his contribution with reference to the "Pathogenetic Organisms," especially in his detailed investigations into the etiology of anthrax, as well as in his studies into the action of disinfectants and germicides made in connection with Wolfhugel. However important these researches may have been, they were but the pioneers that led the way to that discovery which at the time did more to extend Koch's reputation over the world than all his previous work, viz., his researches into the nature of tubercle-virus and the discovery of the bacillus of phthisis. This discovery at first gave rise to the most bitter opposition from various directions, many deeming it absurd or impossible, but when observers became more thoroughly acquainted with the exactness of Koch's method and began to practice it themselves, one objector after another gradually yielded to the supremacy of the greater master, as evinced in his classical contribution to the "Etiology of Tuberculosis," in the second volume of the "Mittheilungen" above mentioned. Scarcely had the excitement begun to quiet down, which the previously considered contributions had made in the minds of both professionals and laity, before Koch again convulsed the world with another discovery, which for the time appears to have absorbed the attention of the whole thinking world, especially of that part of it which is interested in all questions of great public importance.

The government of Germany had selected Koch to conduct the commission which were to proceed to Egypt and India in order to make investigations into the nature and cause of Asiatic cholera. To the enthusiastic zeal, as well as to the simple and yet practical methods of cultivation and research which owe their discovery and completion to Robert Koch, is due the discovery of that most noted parasite, the comma bacillus, which he looks upon as the cause of cholera. It is well known that this discovery has also had the effect of creating as much scepticism and opposition on the part of other observers as was the case with the bacillus of tuberculosis. One can assuredly express the conviction that the same fate will follow Koch's opponents in connection with the comma of cholera, as has been the case with regard to its relation in tuberculosis. In fact, the masterly manner in which he demonstrated and refuted the errors of Finkler and Prior should never be forgotten by those who are busied in the field of bacterial research.

To those whose fortune it is, or has been, to stand in such intimate relations with Koch as to become acquainted with the sceptical criticism to which he subjects all his own work, as well as with the painful exactness of his methods of observation and experiment, it is evident that the contradictory assertions of other observers must be due to insufficient acquaintance with the methodic process of bacterial culture on, or in, solid media.

The discovery and completion of these methods are of themselves sufficient to give the name of Koch a place in the immortal wreath with which Science has graced the world. By means of the same it has become possible for us to search with some degree of certainty for those microscopic fiends which lie at the bottom of many of the most fatal diseases of both animal and vegetable life. I can, therefore, assume that the readers of this JOURNAL will be interested in becoming somewhat better acquainted with the outlines of these methods.

In order that we may assume that a given disease, or disturbance, is due to some micro-organism, it is necessary,

1st. That we find in every case of a like nature an organism having exactly the same morphological characteristics.

2d. That it is possible for us to securely isolate said organism and to cultivate and produce it in the same form, in some artificial media, though several generations outside the organism in which it was first discovered. Only in this way can we be sure that we have finally secured a cultivation absolutely free from any other contaminating material.

3d. We must be able to produce the same disease in an animal selected for the purpose, by means of such a pure cultivation, as the animal from which we procured the original material was afflicted with.

In illustration of the above I have only to mention that Koch has carried cultivations of tubercle bacilli through thirty-four generations outside the animal organism, extending over a period of twenty-two months, and that he was still enabled to produce tuberculosis in a suitable animal at will by inoculations with minimum quantities of said "culture-material," with the same certainty as when the bacilli were first cultivated.

The successful carrying out of any of these conditions is by no means an easy matter in every case. Especially will it be found a question of great difficulty with reference to the first two, when the diseased parts stand in more or less open connection with the outside world, such as the skin, the respiratory, digestive, urinary or sexual organs ; in such cases, numerous adventitious micro-organisms must, of necessity, be present, and the certain isolation of such as may have specific characistics in a questionable case will not be by any means an easy task.

In all such cases, the first task will be the isolation of each individual form of bacterial life, and then to consider and examine them each under the microscope. Before Koch discovered and perfected the method of cultivation by solid media, it was next to an impossibility to successfully isolate the micro-organisms found so frequently infesting diseased organs or tissues. Almost the entire discredit into which the whole doctrine of the pathogenetic fungi has fallen during the last few years must be attributed solely to the failures connected with the previous ways of making investigations into their forms and modes of life which led to erroneous conclusions and unfruitful hypotheses.

It was formerly the custom, and is now, with many observers, Pasteur, Zopf, Nageli, *et al.*—to use fluid media in their cultivations of micro-organisms, such as bouillon and other suitable material, which had been prepared by cooking a sufficient number of times; in this manner all adventitious germs which were previously in the medium were supposed to have been killed. Then was introduced into this fluid a small portion of the suspected material, and in a suitable temperature it could be seen that great quantities of something developed, which, on microscopic examination, were found to belong to germ life. When it accidentally happened that the infectious material—blood, pus, or tissue—contained but one variety of micro-organisms, then it is natural that the cultivating medium, though fluid, would contain but this one form, always providing no other gained access to it during the process of inoculation. This has not generally been found to be the case, however, when fluid media have been used, the same being most frequently found containing a variety of forms of germ life, one form predominating over another according as the conditions of temperature, etc., favored this or that variety. It is evident that experiments made upon animals with pure (?) material gained in this way would be most uncertain and variable in their results. It must be still further borne in mind that in opening and closing the test tubes containing such fluid cultivations, the atrium was given to the entrance of many foreign varieties of germs from the air which could of themselves develop if the conditions were favorable. It was even possible, and not unfrequently probable, that such media could thus acquire pathogenetic properties which it did not possess before the medium had been opened to the free influence of the external air.

This variation in the forms of organisms present in such cultivations led many otherwise most clever observers to the mistaken conclusions that there was but one original, or primary, form of bacterial life, and that there was no such thing as constancy in either their bio- or morphological characteristics among the lower fungi; and that the variation in these directions under which they appeared, were due to variations in the nature of the cultivating media, temperature, etc., and further, it was asserted that a mass of such organisms which to-day were proven to be perfectly harmless could, under change in the above condition, assume on the morrow the most violent pathogenetic characters. The most noted advocates of this idea have been Nageli, Zopf, and Buckner. The views and teachings of Zopf, which are indeed of a most philosophical yet hypothetical nature, may be found in his work on "Die Spaltpilze." They have lately been subjected to a most searching criticism by Flügge of Göttingen in the "Deutsche Medicinische Wochenschrift," 1884, No. 46.

These views with regard to the Polymorphism of the fission fungi have always found an active and determined opponent in Koch. By means of the solid media for the cultivation of these fungi introduced by him, it has been possible to absolutely determine the constancy in form of the organisms in question with the same exactness possible in organisms of a higher type, as soon as we have them before us in the shape of pure cultivations. The contradiction between fluid and solid media is to be sought in the fact that in the first the individual bacteria move about, either actively or passively, through the medium, the separation, or fixing, of the single varieties being thus impossible, while in solid media each individual germ becomes fixed on one locality, from which new individuals develop according to their kind, in the immediate vicinity of the original individuals of their own species, thus forming colonies having exactly the same bio- and morphological characteristics. Each of these colonies retains its individual characteristic so long as it does not become mixed with those in its vicinity, either through progressive development, or too close proximity one to another, or become polluted by adventitious germs falling from the air on removal of the covering glasses. In order to obtain isolated cultivations from any desired material it is only necessary to disperse its individual elements widely enough apart in the cultivating gelatine and to protect the latter from the air.

The following methods are those adopted in the laboratory of the Imperial Board of Health in obtaining pure cultivations of bacteria:*

Koch recommends what he has called "Fleisch-wasser Pepton-gelatine" as the most convenient and general medium for the cultivation of the majority of the pathogenetic fission

* These methods have been described in detail in a brochure by the author, entitled "Uber die Kochschen Reinculturen und den Cholera-Bacillus." Leipzig: 1885.

fungi in the ordinary temperature of our rooms, say 17-19° C. For other germs, which require a higher temperature for their development, say blood-heat, by which the above-named gelatine would become fluid, instead of gelatine, Hesse has recommended an alga, known in Germany as "Agar-Agar" ; such a gelatine remains tolerably solid even at a temperature of 40° C. One can prepare meat-water Peptone-gelatine in a simple manner, as follows :

Take 250 grammes of finely-hashed lean beef, and add to it 500 grammes distilled water ; the same must be placed in an ice-chest, or in the winter in a cool place, and allowed to stand for twelve to twenty-four hours ; it should then be thoroughly stirred up and filtered through a fine cloth ; if the fluid received does not amount to 400 grammes, sufficient distilled water must be added to make it equal ; to this fluid we add 4 grms. Peptonum-siccum (must be pure) 1 per cent.; 2 grms. cooking salt, 1-2 per cent., and 40 grms. of the very best and clearest cooking gelatine, and allow the whole to stand in a vessel for about half an hour until the gelatine has become softened. The mass is then to be put into a flask and thoroughly warmed in a water bath—a common kettle will do in case of necessity—until the gelatine has become completely dissolved, but not sufficiently to cause coagulation of the albumen contained in the meat-juice ; the mass must then be neutralized, it having an acid reaction, by the addition of a saturated solution of pure carbonate of soda, so that very sensitive blue litmus paper will not be changed, and red but very slightly to a delicate blue color.

This is a part of the procedure that requires the greatest circumspection in the preparation of such a gelatine for the cultivation of the comma of cholera asiatica, which will not develop in either an acid or strongly alkaline medium. In the case of the majority of known fission fungi this delicacy of the alkaline reaction is not so necessary, many of them growing luxuriantly in media having a strong reaction of this kind. This reaction, when too strong, can be readily corrected by the addition of a slight quantity of lactic acid.

The deportment of bacteria to different cultivating media is often of much diagnostic value, as many of them grow in a peculiar manner in a medium of known specific reaction. This can only be ascertained by experience.

To return to the preparation of our gelatine material :

The gelatine having been dissolved in a moderate temperature and the reaction corrected, the mass is again placed in a water-bath, the heat increased gradually, and the whole cooked for from one-half to a full hour, until the albumen has been entirely precipitated (this can be readily proven by filtering a small amount of fluid into a test tube and heating it over the burner; if no further precipitation takes place the mass has been cooked enough).

The fluid now loses its redness and becomes slightly straw-colored. The reaction of the mass should be again taken. The whole must now be carefully filtered while hot, in small quantities, great care being necessary not to burst the paper. If the quantity prepared is large, one can place several filters at work at the same time. The filtered fluid should have a clear topaz yellow color, and be at once placed in test tubes which have been previously sterilized, test tubes being filled about one-third. Care must be taken not to touch the mouth of the tubes with the fluid, for on cooling it will cause the cotton plugs to adhere. These tubes must be heated to cooking for about fifteen minutes daily for four or five days, in order to be sure and destroy any germs that may still be present and capable of developing. This can be done either wholesale, by placing the filled tubes in some tin vessel, and then in a bath filled with cold water, which must be heated to boiling; or by heating each tube over a Bunsen burner, care being taken not to cause the fluid to ascend against the cotton, or to boil up so as to form bubbles, or that the tubes dance up and down in the vessel so as to break their bottoms.

These procedures having come to completion, our gelatine is ready for inoculation. To this purpose the gelatine in a test-tube is to be slightly warmed, just enough for it to become fluid. We next take an inoculator, or piece of platinum wire fixed in a glass handle, and heat it to a red heat in a burner, allowing it to cool on touching the point of it into pus, fæces, or any material we suspect as containing malignant or any desired germs, and dip the point into the gelatine, after which we replace the cotton plug and shake up the fluid, in order to disperse the inoculated material through it; we then warm another test-tube in the same way, and after sterilizing our inoculator as before in the gas flame, we introduce it into tube No. 1, and then inoculate tube No. 2, this time four or five times, then shake up its contents. We go through this procedure at least three times, and in this way widely disperse the germs through the medium for cultivation. The gelatine of the last tube is then carefully poured out upon a piece of ordinary window-glass, cut to an appropriate size and previously sterilized. This is placed in a glass dish, in the bottom of which is a piece of moistened blotting-paper, said dish having been previously sterilized; by the above inoculative procedure the germs have been so dispersed through the gelatine that when it cools and hardens on the glass plate, which, being at once covered in the dish, removes all danger of contamination from the air, each germ becomes fixed and isolated where it was at the time the gelatine was poured on the glass, and multiplies at this point, and this only into a colony. Should any germs have accidentally fallen upon the plate while the cover of the dish was off, they also remain fixed, and grow only as an individual cultivation in the place where they fell. The dish is then to be placed on one side and things left to take their natural course.

In one or two days we can see the minute colonies which have developed, with the naked eye; each of these contains but one variety of germ; each has its peculiar kind of development and presents form or color peculiar, more or less, to itself. Some of these bacteria are odorless, while others yield a most offensive smell when the cover of the dish is lifted; others cause the gelatine to become fluid, while others look like dry scales or deposits upon, or in it. If from such an isolated colony, which by microscopic examination we have found to be formed of but one variety of bacteria, we inoculate a test tube, as previously mentioned, and disperse the germs through the warmed gelatine, and then pour this out upon a glass plate as before, we than have growths, or cultivations, dispersed over the surface of the glass, each colony of which consist of one and the same variety of bacteria from both a bio- and morphological point of view. If on the other hand we do not wish to make plate cultivations, but to observe the manner of development of a given bacteria in the solid medium in a test tube, we touch the desired growth upon the plate with the point of the previously sterilized wire, and then, carefully removing the cotton plug from a test tube (which we hold mouth down so that nothing can fall into it from the air, an advantage only offered by solid media), we quickly introduce the glass staff and wire into the tube, pushing the wire into the gelatine or touching its surface—the first is known as a "Stich or puncturing-culture." In this way we are enabled to inoculate from one tube to another as the bacteria consume or destroy the gelatine and thus carry on our cultivations through endless generations, if we desire, the last being exactly like the first, if there are no adventitious germs present. In some cases, while the bacteria suffer no changes either of a bio- or morphological character, they do in their physical attributes, such as weakening in power or virulence, as they pass through so many generations of pure cultivation outside of any animal organism. Bacillus anthracis is illustrative of this peculiarity. By means of this serial cultivation we get the material necessary to the perfection of the last link in the chain of bacterial experimentation; viz., to find out as to the infectious or pathogenetic characteristics of any given germ; *i. e.*, to prove whether it is indeed *the* cause of the disease or processes in question.

By means of these procedures, as previously mentioned, it has been possible for Koch to completely contradict the theory of the polymorphism of bacterial life, and to prove the doctrine that constancy of species is as true of bacterial as it is of human life. Bacillus anthracis can only be produced from its like, and in its turn produce its like, as man can come from and only produce man. This is sufficient to demonstrate the great practical importance of the methodic process perfected by Koch, over that of fluid media, no matter by whom advocated. It needs no arguments to prove that it is the only method upon which we can certainly depend to procure pure cultivations, and to retain them in this condition through any desired number of generations.

The value and importance of these facts are:

1st.—The nature of the medium, or substance, which offers a favorable ground for the growth of any individual variety of bacteria.

2d.—The ability to thus isolate and study it in all its characteristics.

3d.—The fact that they suffer changes in their physical characteristics, as has been shown, or better indicated by Pasteur for quite a number of germs.

These, and other facts yet to be ascertained, all go to show the great value of Koch's perfection of solid cultivating media, both for the treatment and prevention of contagious and infectious diseases. It should here be mentioned that by means of his simple method, Koch was enabled to show that the bacillus which Prior and Finkler discovered in the evacuations of cholera nostrus, and which they claimed to have cultivated in a pure form, and to be similar in all respects to that discovered by Koch in cholera asiatica, was not, in their cultivation, a single germ, but rather that these observers had been cultivating a mixture containing four varieties of bacteria, each havingdistinct morpho and biological characteristics, to show the great difference which existed between the comma found by him in cholera asiatica and that really existing in the cultivations from cholera nostrus by Prior and Finkler. As to the morphological variations between these two commata, it is not to be denied that when embedded in Canada balsam, they present a very close resemblance; but this apparent correspondence vanishes when the practised observer examines freshly colored specimens at once in water, with the same microscope and same power. It can then be easily seen that the comma of Koch is exceedingly thin and delicate, while that of Finkler is larger and thicker—that is, more plump. If, on the contrary, we should admit that morphologically the two varieties presented no ascertainable differences perceptible with our present means of ocular examination, still it does not follow that the biological conditions should so closely correspond, any more than that the chemical characteristics and action of a sweet and bitter almond should be the same, no matter how nearly alike the outside appearance may be. Notwithstanding this apparent morphological similarity between the Koch and Finkler commata, their biological phenomena are essentially different.

The comma of Finkler and Prior demonstrates much greater activity in proliferation, under like conditions of cultivating media and temperature, than that of Koch. When cultivated in gelatine upon the glass plate, the single colonies of the Finkler and Prior comma present a fine granulated appearance when examined by a low power under the microscope, and one of a homogeneous round form and have a distinctly yellowish brown color; they also cause the gelatine to become fluid very quickly and develop a most penetrating and intensively unpleasant odor in the vessel used for cultivating them, so that in from twenty-four to thirty-six hours they have sunk deeply into the substance of the gelatine, and caused quite an area of fluid degeneration in the vicinity of each colony. The colony soon loses its finely granulated and circumscribed appearance, becoming very irregular in form. It causes fluid-degeneration of the gelatine so rapidly that in the course of two or three days the whole mass upon the glass plate has become destroyed.

The commata of Koch present quite other characteristics; their colonies, under exactly the same conditions, developing much more slowly and *never* presenting a brown color. When fresh they have a delicate yellowish red color. They do not cause any such rapid fluid-degeneration of the gelatine in their immediate vicinity; the culture gradually sinks into the substance of the gelatine, forming a sort of funnel-shaped excavation, at the bottom of which may be seen the bacteria as a small dark point. Viewed with the microscope such colonies do not present the sharply-marked, smooth-edged circumferences of the Finkler comma, but rather jagged or indented edges, as if the whole colony consisted of a collection of finely-powdered glass particles; the offensive gases developed by the Finkler are not produced by the Koch commata, even after the elapse of five or six days, although such cultures give off an odor somewhat resembling that of urine. The contrast in the morphology of the growths of these two germs is still greater when observed in puncture cultivations in gelatine in tubes. The accompanying woodcuts show these variations in a very characteristic manner, and have been approved by Dr. Koch.

Even the layman can at once see the energetic development which occurs in the Finkler, in from two to four days, in comparison with the Koch, whereby the former causes very rapid fluid-degeneration of the gelatine, not only on the exposed surface but during the entire length of the puncture in the gelatine; in the one case the Finkler has the form of a pout-leg or "cordon," while in the other it is more like a mushroom. By the Koch comma this degeneration takes place much less rapidly and not in the entire length of the puncture at once, but extends very gradually.

These fungi also present equally characteristic biological variations when cultivated upon cooked potatoes. While the Finkler develops luxuriantly upon this medium in ordinary room temperature, forming a colony of a greyish yellow color, encircled by a whitish zone, the Koch comma only grows upon this material at a blood temperature, say 37° C., and forms dark brown colonies similar to those presented by those of glander bacilli.

It must be especially noted that these variations in the biological conditions presented by these two fungi are not by any means accidental, but are, rather, a constant phenomena, as has been sufficiently proven by hundreds of comparative cultivations in the Laboratory of the Imperial Board of Health, as well as by other observers, especially Ermengem of Brussels.

Koch has also proved the same thing in connection with a comma found in the oral cavity by Lewis (*Lancet*, Sept., 1884, p. 513). His bacterium was known long before Lewis discovered it, as shown by Miller of Berlin (*Deutsch. Med. Wochenschrift*, 1884, No. 48), and the morphological similarity is not so close as claimed by Lewis; on the other hand, there are no biological resemblances between this commata and that of Koch; the former does not develop at all in either a neutral or slightly acid gelatine.

So far as the evidence at our command may be at present summed up, it goes to show that the comma of Koch has specific connection only with Asiatic cholera, no one, not even Finkler and Prior, having been able to discover any other form of comma bacteria with precisely the same morpho- and biological characteristics as that of Koch, or which could by any means be taken for the latter when cultivated according to the strict letter of the law as demanded by Koch's methodic process.

The value of this fact cannot be over-estimated when taken in connection with the Etiology and Diagnosis of cholera, or choleroid diseases, as may be at once seen by comparing our illustrations.

It can be most dogmatically asserted that even though the etiological connection between Koch's comma and Asiatic cholera still remains to be proven by direct experiment, still the cultivation of this and morphologically similar bacteria has, thus far, invariably shown the absolute value of Koch's comma as a means to the correct differentiation between two given diseases, and the sure diagnosis of Asiatic cholera.

So much cannot be said in favor of either the assertions or discoveries of any of Koch's opponents. Finkler and Prior

(or somebody else) have got to show that the comma discovered by them is even a constant phenomenon in connection with cholera nostrus, or that it has any differential diagnostic value for this last disease.

Summing up, then: My experience during the cholera courses at Berlin has led me to conclude:

1st. That the comma of Koch is a micro-organism peculiar to cholera Asiatica, and that the demonstration of its presence in the discharges, or in the intestinal contents, of a given person, is a sure means to the diagnosis of this disease.

2d. That the biological variations between this comma and others in gelatine cultivations are also so marked as to be of essential, and, at present, of absolute diagnostic value.

## Figures and Tables

**Figure f1:**
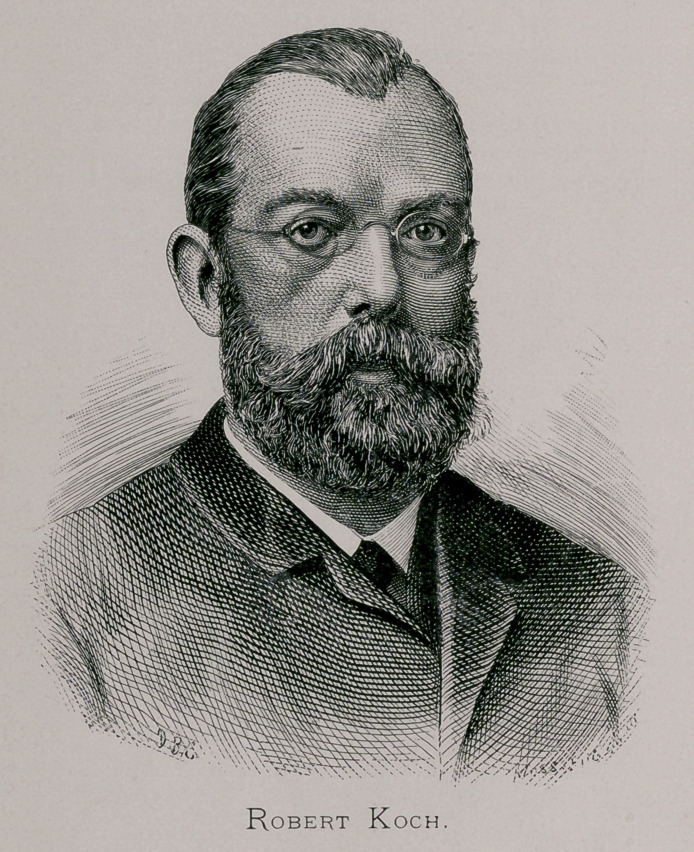


**Figure f2:**